# Sunscreen prevention of melanoma in man and mouse

**DOI:** 10.1111/j.1755-148X.2010.00756.x

**Published:** 2010-12

**Authors:** Heather L P Klug, Janet A Tooze, Cari Graff-Cherry, Miriam R Anver, Frances P Noonan, Thomas R Fears, Margaret A Tucker, Edward C De Fabo, Glenn Merlino

**Affiliations:** 1 Regional Academic Health Center-Edinburg, The University of Texas Health Science Center at San AntonioEdinburg, TX, USA; 2 Department of Biostatistical Sciences, Wake Forest University School of Health SciencesWinston-Salem, NC, USA; 3 Laboratory Animal Science Program, SAIC-Frederick, IncNCI-Frederick, Frederick, MD, USA; 4 Department of Microbiology, Immunology and Tropical Medicine, School of Medicine and Health Sciences, The George Washington University Medical CenterWashington, DC, USA; 5 Division of Cancer Epidemiology and Genetics, National Cancer InstituteNIH, Bethesda, MD, USA; 6 Laboratory of Cancer Biology and Genetics, National Cancer InstituteNIH, Bethesda, MD, USA

Dear Sir,

Cutaneous malignant melanoma incidence has more than doubled over the past 25 yrs, and this trend continues across all age groups at a rate of over 3% per yr (Linos et al., 2009). Exposure to the ultraviolet (UV) portion of sunlight is strongly implicated in melanoma etiology and considered the major environmental risk factor. Higher melanoma risk is associated with burning UV doses, both intermittent and during childhood (Gandini et al., 2005). Alarmingly, melanomas are the most prevalent cancer in 25–29 yr old females, and a link to commercial tanning sunlamps use is suggested (Purdue et al., 2008). Melanoma prevention recommendations include avoiding mid-day sun and artificial UV light, wearing protective clothing and hats, and using sunscreen with a Sun Protective Factor (SPF) of 15 or higher (American Cancer Society, 2010). However, the SPF sunscreen rating describes erythema or sunburn protection; sunscreens are not rated for the prevention of melanoma or other skin cancers (FDA, 1999). Melanoma incidence reduction through sunscreen use has not yet been proven, and in fact has been controversial (Saraiya et al., 2003). We here present data that demonstrate for the first time significant sunscreen prevention in UV-dependent, melanoma-prone transgenic mice, and propose that when applied properly should be preventive in people.

Human epidemiologic studies of melanoma prevention are limited by recall bias, insufficient statistical power, and non-uniform estimations of sun exposure and sunscreen use. Therefore, sunscreen use and melanoma risk reduction or mortality prevention are difficult to accurately assess and remain inconclusive (Bastuji-Garin and Diepgen, 2002; Saraiya et al., 2003). We evaluated sunscreen use as a risk factor for melanoma in a large case-control study with 717 non-Hispanic white, invasive melanoma patients and 945 matched controls (Fears et al., 2002). The univariate analyses of ever sunscreen use or regular use of sunscreen of SPF ≥ 8 indicated minimal risk associated with sunscreen use (relative odds 1.05 and 1.11, respectively). This association is suggested to reflect users’ sun sensitivity (burning and tanning tendencies), and not a property of sunscreen exposure per se (Dennis et al., 2003). A matched logistic regression analysis of sunscreen use adjusted for average UVB intensity of residences, adult hours outdoors, tan-type, number sunburns, age-group, study-site and gender (Appendix S1), showed that sunscreen use was not associated with melanoma (OR 0.90, 95% CI 0.70–1.16; Table 1). When evaluating sunscreen users and examining risk in those who burned easily and persons who did not burn in a single model, the relative odds for those who burned easily decreased slightly to 0.85 (0.62–1.19) and for those who did not burn was 0.91 (0.70–1.19) with no statistically significant difference in risks (Table 1). The changes in risks observed after appropriate adjustment likely reflect confounding, a spurious relationship, as persons using sunscreens may be more susceptible to melanoma. These data demonstrate the challenges of assessing complex behaviors, such as sunscreen use, outside of a prospective randomized study. Even in our large investigation, difficulty in addressing these challenges illustrates the urgent need for appropriate animal models, employed experimentally, to better assess the value of sunscreen use in melanoma prevention.

**Table 1:** Melanoma in persons with no sunscreen use, and those who used any sunscreen, among sunscreen users by tendency to burn or sun sensitivity

Sunscreen use	Number of controls^a^	Number of cases^b^	Total	OR^c^ (95% CI)
No use	202	147	349	Referent
Ever use	743	570	1313	0.90 (0.70–1.19)
Total	945	717	1662	
Burn easily	186	160	346	0.85 (0.62–1.19)
Do not burn	557	410	967	0.91 (0.70–1.19)
Total	743	570	1313	

a Persons recruited from same geographical area as Cases, and matched on age, sex, race.

b Persons with histologically confirmed cutaneous malignant melanoma diagnosis.

c All OR adjusted for ambient residential UV intensity, number of hours outdoors, tan type, number of sunburns, gender, age group, and study site.

The availability of the Hepatocyte Growth Factor/Scatter Factor (HGF/SF) genetically engineered mouse, which develops neonatal UV-dependent skin lesions highly reminiscent of cutaneous malignant melanoma (Noonan et al., 2001), provides a favorable platform to demonstrate experimentally the capabilities of sunscreen in a melanoma prevention study. This mouse model has been used to support the notion that childhood sunburn constitutes a major melanoma risk factor (Noonan et al., 2001). Therefore, we hypothesized that blocking UV radiation-induced erythema with current dermatologist recommended SPF15 sunscreen would significantly decrease the incidence of melanoma in this relevant murine melanoma model.

Neonatal mice were exposed to a single dose of UV radiation with prior application (15 min) of either vehicle-control lotion or SPF15 sunscreen containing FDA-approved active agents (Appendix S1). The UV dose corresponded to a human erythemally-weighted UV dose of 2.3 kJ/m^2^ or 23 Standard erythemal dose units, equivalent to a sun-burning dose in people (Noonan et al., 2001). The primary endpoint was melanoma development in the dorsal application area, confirmed by histopathology and positivity for melanocytic antigens. The SPF15 sunscreen-treated animals developed significantly fewer melanomas than the vehicle-control group (P = 0.043; Figure 1). In the sunscreen-treated group (n = 97), one mouse (1%) developed two dorsal melanomas, while the eight mice (7%) in the vehicle-control group (n = 118) developed 18 melanomas (Figure 1; Table S1). The multiplicity of melanomas per animal arising in the sunscreen-treated group was also lower, with two per animal versus up to seven per animal in the vehicle controls. Animals from both groups equally developed melanoma outside of lotion-treated areas (tails and/or hindquarters): two in vehicle controls and one in the SPF15 group.

**Figure 1:**
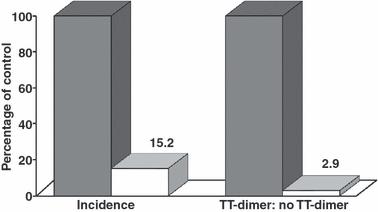
UV-induced melanomas and UV-induced DNA damage are significantly inhibited in sunscreen-treated compared to vehicle control-treated mice. Melanoma incidence per group and the average ratio of TT-dimer-containing cells to no TT-dimer-containing cells are presented as a percentage of control. Filled bars, vehicle control-treated mice; open bars, SPF15-treated mice. The numbers indicate the percentage of control for the SPF15 group incidence, and for the SPF15 group TT-dimer ratio.

The molecular mechanisms by which sun and artificial UV light initiate and/or promote melanoma development are being elucidated (Zaidi et al., 2008). Clearly, both direct and/or indirect DNA damage mechanisms, including thymine dimer and genotoxic reactive oxygen species formation, may play a mechanistic role. We therefore quantified DNA damage in skin cells of UV-treated control and SPF15 sunscreen-treated animals using an antibody to thymine-thymine (TT) dimers, a type of damage initiated by UVB irradiation. Cells were scored ‘positive’ if double-stained for TT dimers and nuclear counter-stain at seven minutes post-UV. The vehicle control lotion-treated skin averaged greater than five cells with nuclear DNA damage to each non-DNA damaged cell (Figure 2; Table S1). In sunscreen-treated skin, the TT:no-TT ratio was <1, indicating a distinct sunscreen protective effect in UV-exposed skin (P = 0.004; Figure 2; Table S1). The UV-induced DNA damage in vehicle control lotion-treated skin extended throughout the length of the epidermis and into dermis and upper hair follicle regions (Figure 2A and inset; anti-TT positive, brown nuclei; white arrow). In contrast, only small patches of DNA damage-containing cells (Figure 2B, white arrow) were observed along the epidermis and epidermal/dermal junction of SPF15 sunscreen-treated animals (Figure 2B and inset; blue counter-stained nuclei; yellow arrow). Thus, a sunscreen protective-effect against UV-induced damage is observed in the treated animals’ skin.

**Figure 2:**
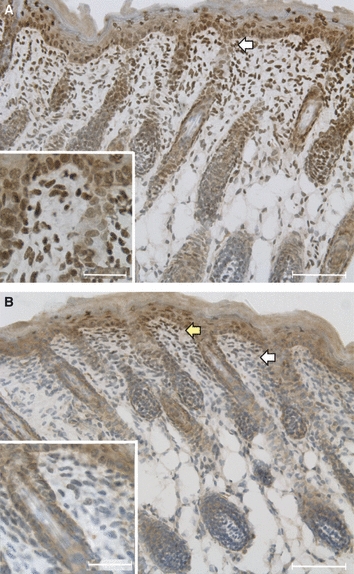
Sunscreen use decreases UV-induced DNA damage in the skin of mice. Skin micrographs are representative of all those observed; tissues were harvested at 7 min post-UV irradiation. (A) Throughout the skin of a control lotion-treated animal, brown nuclei, positive for anti-TT dimers, are found. The white arrow highlights brown nuclei, and the area of the inset photo. (B) In skin from a SPF15-treated animal, a typical area shows blue-grey nuclei with no or few TT-dimers (white arrow; area of inset photo). A rare patch of brown, anti-TT reactive nuclei is observed in the epidermis/upper skin (yellow arrow). Scale bars in (A) and (B) are 50 micrometers. Scale bars in both (A) and (B) insets are 20 micrometers.

The major environmental risk factor for melanoma is well established to be UV exposure. Further, cumulative UV exposure, both in childhood and as adults, contributes to melanoma etiology and its’ expanding epidemic (Lea et al., 2007; Linos et al., 2009; Tucker and Goldstein, 2003). We employed a relevant animal model of cutaneous malignant melanoma to corroborate, for the first time, what present and previous epidemiological data has only suggested but not proven: sunscreen use can significantly inhibit melanomagenesis. In addition, we correlate sunscreen use in the same mouse model with prevention of DNA damage in UV-irradiated skin. Our data advance arguments for a public health strategy to prevent melanoma and reduce mortality involving sunscreen use, as well as UV-irradiation avoidance behaviors and increased access to screening.

## Supporting information

**Table S1.** Sunscreen use decreases UV-induced melanoma development and DNA damage in HGF. SF transgenic mouse skin.
