# Supplementary material for: Sunscreen prevention of melanoma in man and mouse
Source: Pigment Cell Melanoma Res. 2010 Dec;23(6):835–7. doi: 10.1111/j.1755-148X.2010.00756.x (PMC2995311; doi:10.1111/j.1755-148X.2010.00756.x)
Supplement: Supplementary file 1 [file pcmr0023-0835-SD1.doc]

**Supplementary Table S1.** **Sunscreen use decreases UV-induced melanoma development and DNA damage in HGF/SF transgenic mouse skin.**

| **Treatment**  **(txt)** | **N** | **Animals with melanoma within txt area*** | **Multiplicity within txt area**† | **Ratio of TT-dimer to No TT-dimer skin cells**§ | **Median Days at Risk** | **Mean Days at Risk** |
| --- | --- | --- | --- | --- | --- | --- |
| **Control** | 118 | 8 (0.07) | 18 | 5.40 | 171 | 252 |
| **SPF15** | 97 | 1 (0.01) | 2 | 0.16 | 133 | 246 |

******P*=0.043 by Fisher’s exact test; percentages are shown in parentheses.

†One control animal each had 7, 4 and 2 melanomas; five animals had 1 melanoma. One sunscreen-treated animal had 2 melanomas. *P*=0.094 by Fisher’s exact.

§*P*=0.004 by two-sample Wilcoxon rank-sum test. Background ratio, No-anti-TT antibody used, is 0.014.
