# Supplementary material for: Sunscreen prevention of melanoma in man and mouse
Source: Pigment Cell Melanoma Res. 2010 Dec;23(6):835–7. doi: 10.1111/j.1755-148X.2010.00756.x (PMC2995311; doi:10.1111/j.1755-148X.2010.00756.x)
Supplement: Supplementary file 2 [file pcmr0023-0835-SD2.doc]

**Appendix S1. Materials and Methods**.

**Materials and Methods:**

**Human Study Design and Analysis.** Patients, ages 20-79, with histologically confirmed cutaneous malignant melanoma were recruited in a clinic-based case-control study at the University of Pennsylvania and the University of California at San Francisco from Jan. 1991-Dec. 1992, as previously described (Fears et al., 2002, Tucker et al., 1997). Controls from the same geographic area as cases were matched on age, sex, and race to melanoma cases. Participants were interviewed in person by trained interviewers. Average UVB intensity of residences for participants were constructed as described (Tucker et al., 1997). At the time of this study, sunscreens were generally not broad spectrum and did not contain vitamin A or its derivatives. Fewer than 10% of individuals in the study used SPF15 or greater sunscreens.

**Animals.** The HGF/SF transgene is expressed in FVB/N inbred, albino mice under the control of the metallothionein gene promoter. Animals were maintained in accordance with NCI/NIH institutional guidelines and AAALAC approval.

**Animal Treatment.** Lotions were applied at 2 mg/cm^2^ and according to FDA guidelines for sunscreen Sun Protection Factor (SPF) assessment (FDA, 1999) in the vehicle control and SPF15 treatment groups, including a 15 min waiting period prior to UV exposure. The ingredients of lotions (Cosmetech Laboratories, Inc., Fairfield, NJ) in the two groups were identical except for the use of sunscreen actives in the SPF15 lotion. The base formula contained water, sorbitol, triethanolamine, methylparaben, propylparaben, lanolin, cocoa butter, cerasynt Q, stearic acid, and benzyl alcohol. The SPF15 sunscreen actives were benzophenone-3 (UVB/UVA absorber) and Escalol 507 (UVB absorber). Matching the human study, the lotions did not contain vitamin A or its derivatives. Sunscreen active ingredient use and SPF rating determination followed FDA guidelines for sunscreen development and testing (FDA, 1999). The average SPF rating was 1.09 for control lotion and 15.39 for SPF15 lotion. Trial experiments were done to ensure pups would be accepted back by the dam after lotion and irradiation treatment. UV irradiation of HGF/SF transgenic mice was carried out as previously described (Noonan et al., 2001, De Fabo et al., 2004).

**DNA Damage Assessment and Histology.** Two control FVB/N wild-type mice and two SPF15 FVB/N wild-type mice were irradiated as described above, and skins harvested and frozen (liquid nitrogen) at 7 min post-UV. Sections were stained for thymine-thymine (TT) dimers (clone KTM53, Kamiya Biomedical Co., Seattle, WA) and with hematoxylin (Tadokoro et al., 2003). Over 400 cells in each field of view were scored as ‘positive’ if double-stained for TT-dimers and hematoxylin. Positive cells in 3 slide fields per mouse were counted, and a TT-dimer to no TT-dimer cell ratio was calculated. An average TT-dimer to no TT-dimer ratio was calculated for each treatment group (Table S1). Photomicrographs were taken using a DFC280 Leica camera on a DMI6000B Leica digital microscope using Leica Application Suite software (version 3.5).

**Animal Study Data Analysis**. Statistical analyses were performed using Stata 10 software (College Station, TX). The Wilcoxon rank sum test was used for comparisons of continuous data and a Fisher’s exact test was used to compare incidence rates between groups. All tests were two-sided with an alpha-level of 0.05.

**Materials and Methods References**

De Fabo, E.C., Noonan, F.P., Fears, T. & Merlino, G. (2004), Ultraviolet B but not Ultraviolet A Radiation Initiates Melanoma. Cancer Res*,* *64*, 6372-6376.

FDA, DHHS (1999), Sunscreen Drug Products For Over-The-Counter Human Use; Final Monograph. DHHS, FDA, ed. Federal Register.

Fears, T.R., Bird, C.C., Guerry, D.T., Sagebiel, R.W., Gail, M.H., Elder, D.E., Halpern, A., Holly, E.A., Hartge, P. & Tucker, M.A. (2002), Average midrange ultraviolet radiation flux and time outdoors predict melanoma risk. Cancer Res*,* *62*, 3992-6.

Noonan, F.P., Recio, J.A., Takayama, H., Duray, P., Anver, M.R., Rush, W.L., De Fabo, E.C. & Merlino, G. (2001), Neonatal sunburn and melanoma in mice. Nature*,* *413*, 271-2.

Tadokoro, T., Kobayashi, N., Zmudzka, B.Z., Ito, S., Wakamatsu, K., Yamaguchi, Y., Korossy, K.S., Miller, S.A., Beer, J.Z. & Hearing, V.J. (2003), UV-induced DNA damage and melanin content in human skin differing in racial/ethnic origin. FASEB J*,* *17*, 1177-9.

Tucker, M.A., Halpern, A., Holly, E.A., Hartge, P., Elder, D.E., Sagebiel, R.W., Guerry, D.T. & Clark, W.H., Jr. (1997), Clinically recognized dysplastic nevi. A central risk factor for cutaneous melanoma. JAMA*,* *277*, 1439-44.
